# Cohesive Zone Modeling of Lap Shear Behavior of Different Metal Alloys and Glass-Fiber-Reinforced Thermoplastic Polyamide Hybrid Composites

**DOI:** 10.1021/acsomega.6c00568

**Published:** 2026-05-04

**Authors:** Mete Kayihan, Yasemin Certal, Mustafa Bakkal

**Affiliations:** † Mechanical Engineering Faculty, Istanbul Technical University, Istanbul 34467, Turkiye; ‡ Turkish Aerospace Industries Inc., Mechanical Engineering, Istanbul 34906, Turkiye

## Abstract

There is an increasing tendency toward adhesively bonded hybrid structures including thermoplastic matrix composites and some metallic alloys. In this context, this study aims to investigate the mechanical properties of adhesively bonded single-lap joints (SLJ) both experimentally and numerically. Therefore, a series of lap shear tests and numerical analyses are conducted on 35% glass-fiber-reinforced polyamide, aluminum, titanium (Ti_6_Al_4_V), and stainless-steel (SS304) alloys hybrid bonded joints. For determination of the effect of design parameters on joint strength, SLJ tests are carried out on specimens with different thicknesses, composite sequences, and overlap lengths. Moreover, the adhesive and bonded specimen interface is modeled using cohesive zone modeling (CZM). After examination across three different hybrid materials, the CZM model yields largely similar results to the experimental tests. The highest similarity is achieved with SS304, with a difference of only 1.5% with 6.1 ± 0.05 MPa. Also, the lowest similarity can be seen with Ti_6_Al_4_V, with a difference of 15% with 5.4 ± 0.06 MPa. Additionally, it is observed that laser surface texturing after sandblasting provides higher shear strength than abrasive sanding and sandblasting by creating a lap shear strength of 14.7 ± 0.18 MPa.

## Graphical abstract

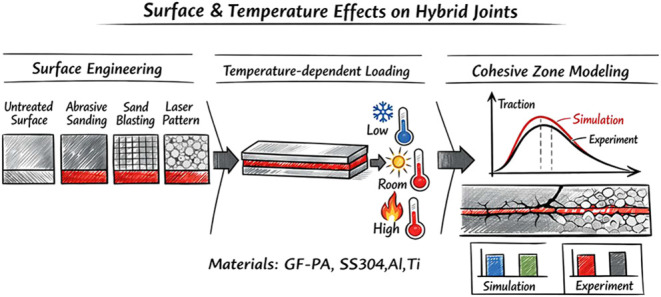

## 1 Introduction

With the advancement of technology and industry, light-weighting efforts have also gained focus in the aviation sector.^1^ The usage of adhesives and sealants relates to technical improvements made in the aviation and aeronautical sectors, as achieving a lower weight for aircraft and spacecraft is a major goal.

Metals are fundamental materials for many industries such as automotive, aerospace, and construction. One of the difficulties encountered in light-weighting efforts is the need to combine different types of metals with different composite materials. Furthermore, some metals cannot be welded, or the strength of the welded metals may decrease significantly. For instance, aluminum alloys have disadvantages related to weldability due to their chemical composition, and even if the alloys are suitable for welding, they must be compatible. In such cases, mechanical fastening is often preferred due to standard practices, products, and well-known potential failures.^2^

Alternatively, adhesives are the most commonly used materials in joining aluminum, titanium, and other metal materials used in aircraft today, especially in composite tails in the aviation industry. Furthermore, drilling holes into different types of materials, while quite popular, can lead to damage in composite material such as delamination and fiber breakage, thus reducing fatigue strength.^2,3^ Additionally, some of the reasons for preferring bonding over welding include the absence of a decrease in strength behavior due to the heat-affected zone, the ability to accommodate larger tolerances, diminishing the possibility of corrosion, boosting aerodynamic effect, a more aesthetic appearance, and a reduction in the manufacturing cost of the structure.^4^

For controlling adhesive bonding, surface energy, mechanical interlocking, and interfacial interactions work together. Classical adhesion theories state that sufficient wettability and close contact between the adhesive and adherend surfaces are necessary for successful bonding. Furthermore, material characteristics and surface shape, which dictate stress transfer and damage evolution mechanisms, affect interfacial fracture behavior.^5,6^ Cohesive zone concepts, which link traction-separation behavior to energy dissipation during crack propagation, are useful for describing interfacial fracture and adhesion behavior. By connecting cohesive modeling, adhesion performance, and surface morphology, the current work expands on these theoretical underpinnings.

There are also many manufacturing processes based on newly developed adhesive bonding methods. These methods are used to improve on traditional methods or to provide additional benefits. These are generally laser textured, weld bonding, hybrid riveting, and hybrid punching.^7,8^ Joining dissimilar materials using welding is particularly prevalent. In adhesively bonded metal-composite hybrid structures subjected to shear stress, damage caused by the adhesive can occur in the form of adhesive slip, cohesive slip, adhesive peeling, or cohesive separation.^9,10^ Adhesively bonded hybrid structures can be varied in terms of the relative positions of the adhesive and adherends, depending on the requirements of the design and the expected load. Single-lap bonds are frequently used in adhesive testing of steel between composite materials.^11,12^

The reliable and efficient use of adhesively bonded joints is dependent on the design and methodology. Finite element analysis plays a crucial role in achieving an optimal structural design. Using the finite element method, accurate predictions of joint strength and fracture behavior can be obtained. Damage mechanics is a method used to predict both initiation and propagation in a structure, up to the full extent of structural failure and fatigue.^7,13^ It can be divided into two main categories: the local approach and the continuum approach. The local approach is used to predict interfacial failure between two surfaces. The interface elements are modeled by using a zero-volume line. The continuum approach uses finite-thickness elements to simulate the failure of a bulk material (adhesive). A specific model called the cohesive zone model (CZM) is categorized between these two approaches and is also used for both two composites and two different materials.^14–20^ The CZM is used for paths defined in the local and continuum approaches and combines the traction-separation response to simulate crack initiation and propagation.

This study addresses an existing gap in the literature by examining the mechanical performance of a glass-fiber (GF)-reinforced polyamide thermoplastic composite material bonded to three different metallic materials (aluminum, stainless steel, and titanium) from a multifaceted perspective. Furthermore, it creates a baseline untreated surface between surface treatment, experimental lap shear behavior, temperature effect, and cohesive zone modeling, in contrast to traditional studies that handle surface preparation and numerical modeling independently. A more accurate depiction of interfacial behavior is made possible by the integration of various elements into a single framework, which is the primary contribution. Furthermore, the cohesive parameters are calibrated based on experimental findings for various surface conditions rather than being just adopted from the literature, which improves prediction capability. Surface morphology, adhesion performance, and cohesive modeling parameters have frequently not been systematically correlated in existing research. Compared with traditional techniques, this integrated approach offers a more thorough understanding of adhesive joint activity. Experimental investigations of the effects of three different surface preparation methods on bond strength provide critical practical data for optimization, while lap shear tests support these theoretical approaches with concrete mechanical data. Moreover, to validate these experimental findings and provide predictive capabilities, separate CZM-based finite element analyses developed for three different material combinations offer a novel approach and provide a reliable predictive tool for future designs. This triangular structure (surface preparation, experimental testing, and advanced modeling) provides a valuable contribution to materials science and adhesion mechanics, bringing about both methodological and scientific innovation. At the same time, this study is of critical importance in order to determine and predict which metal will be used with this composite material in the industry.

## 2 Materials and Methods

This section analyzes the single-lap hybrid configurations constructed from different structural materials, specifically Al6061 T6 aluminum alloy, AISI 304 stainless steel, Ti_6_Al_4_V titanium alloy, and PA66 35% GF-reinforced composite material. These adherents were joined using Hysol Loctite EA 9394, an epoxy-based adhesive system.

The 2 mm thick metallic materials, Al6061 T6 and AISI 304, were supplied from Birçelik (Istanbul). Moreover, the annealing of Al6061 was performed at the Istanbul Technical University Mechanical Engineering Faculty Metallography Laboratory. The Ti_6_Al_4_V was procured from Turkish Aerospace Industries (Ankara). The 2.5 mm thick PA66–35% GF-reinforced composite material used in the tests was supplied by Farplas Inc. under the DuPont Zytel brand and exhibits characteristics crucial for high-performance applications. Its high strength, toughness, and hardness, along with its resistance to abrasion and chemical attack, make it suitable for use in hybrid joint structures in the aerospace and automotive industries. The mechanical and physical properties of the materials are listed in Table 1.

**Table 1:** Mechanical and Physical Properties of the Materials

materials	Al6061 T6	AISI 304	Ti_6_Al_4_V	PA66 35% GF
density (g/cm^3^)	2.7	8	4.43	1.4
tensile strength (MPa)	310	505	920	140
elongation (%)	20	70	10	5
elastic modulus (GPa)	68.9	193	110	8
shear modulus (GPa)	26	74	44	86

### 2.1 Surface Preparation for Adhesive Bonding

Cleaning and preparation of the surfaces of metal and composite parts were carried out according to the international surface preparation standard (ISO 17212).

For reproducible adhesion performance, samples precleaned by spraying isopropyl alcohol from a pressurized plastic bottle onto the surface are soaked in detergent water at approximately 80 °C for 20 min for removing organic residues. Samples taken from the beakers are then placed back into the isopropyl bath and rinsed with distilled water to ensure complete removal of surface impurities. Polyamide materials are known to absorb moisture from the environment, which can affect the adhesion and mechanical performance. For minimizing moisture effects, the samples are dried for at least 20 min using a heat gun with air at 65 °C after cleaning steps. The cleaned sample surface is abraded with 60-grit sandpaper for metals and composite materials to enhance surface activation and promote mechanical interlocking. Following this surface roughening process, particles and dust detached from the surface are cleaned with a distilled water jet. In tests conducted with samples roughened using coarse sandpaper (60 grit), although the adhesion properties of the surfaces were relatively good, the sandblasting method was tried due to the difficulty of obtaining a homogeneous surface for surface roughening. Laser surface texturing processing was applied after sandblasting to achieve a controlled surface morphology. The sandblasting process gives a chance to remove surface contaminants and native oxides. The subsequent laser surface texturing creates well-defined microscale features on a clean and uniformly roughened surface. This step-by-step procedure enhances mechanical interlocking and adhesion performance. The laser surface texturing was applied in the form of perpendicular lines at a 45° angle to the tensile direction of the sample. Each line had a depth and width of 0.5 mm. The laser surface texturing parameters are 40 W power, 200 mm/s velocity, 35 kHz frequency, and 20 pass number. The micrographs of surface preparation techniques are given in Figure 1.

**Figure 1:**
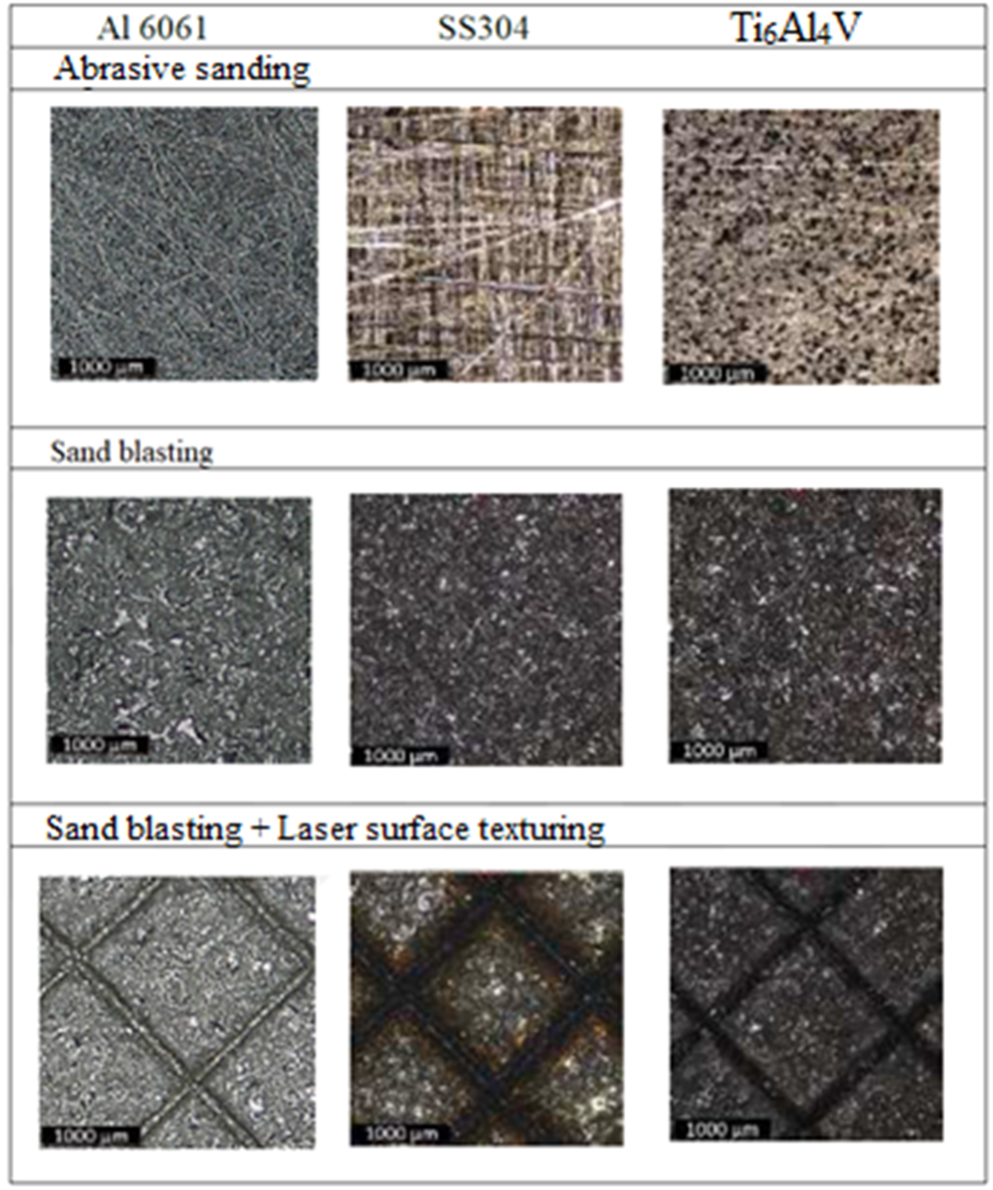
Micrographs of metal surfaces exposed to different abrasion methods.

The directional surface of the PA66 composite material, resulting from the production technique, was eliminated by sandblasting to create a direction-independent surface. When the images are interpreted together with the roughness values, it can be seen that the pits created by the sandblasting process form crater-like structures. Surface roughness is a parameter that is directly proportional to the adhesion performance and slip resistance of the adhesive bond.

Proper curing and uniform application of the adhesive to the surface are as important as thorough surface cleaning for the adhesive to bond correctly and successfully. The adhesive must be applied at a specific thickness during curing to allow polymer bonds to form between the surfaces and achieve the desired strength. The application thickness of the Hysol Loctite Aero 9394 adhesive was targeted at 1 mm. The adhesive was applied uniformly to the bond surface using a spatula to ensure complete wetting. During assembly, calibrated 1 mm diameter glass beads were used as spacers to maintain a precise bond thickness under clamping pressure. After curing, the bond thickness was checked at numerous locations, confirming a mean thickness of 1 mm with a uniform joint thickness.

The adhesive joints were first left at room temperature (23 °C) under controlled laboratory conditions for 1 h to prevent adhesive flow and to ensure uniform adhesive thickness. Subsequently, they were postcured in an oven at 85 °C for 1.5 h to achieve optimal mechanical properties. Afterward, the sample is left to cool slowly in the oven. Additionally, our samples were conditioned in a climate chamber at +85 °C for 48 h and at −40 °C for 24 h to expose the adhesive joints to elevated and subzero temperature conditions, in accordance with the ASTM D1151 standard.^21^ They were then removed from the chamber and tested in a tensile testing machine as quickly as possible without allowing the samples to undergo any temperature changes. Moreover, a standard fixture was designed to maintain the alignment of the two parts being bonded during the bonding process and to ensure that all bonding occurs under equal load and temperature conditions.

### 2.2 Lap Shear Testing

One of the most commonly used test methods for testing adhesive joints is the lap shear test on single-bonded joints. Conducted in accordance with the ASTM D1002 standard, the test involves bonding samples consisting of three different metallic parts (aluminum, titanium, and stainless steel) and a thermoplastic composite material at a single point and then applying a tensile test in the shear direction to the bonded area.^22^ Tensile tests were carried out using a Shimadzu 5 kN tensile testing machine at the Mechanical Testing Laboratory of the Faculty of Mechanical Engineering at Istanbul Technical University. The tensile speed was selected as 1.3 mm/min, as specified in ASTM D1002. Each test was repeated three times.

The metals used in the single-layer adhesive bonding test consisted of 2 mm thick Al6061 T6, AISI 304 stainless steel, and Ti_6_Al_4_V sheets. These metals were bonded to PA66 composite parts reinforced with 35% GF for the purpose of developing the best hybrid bonding alternatives. Figure 2 shows the representation of the paste in panel (a) and the experimental setup in panel (b).

**Figure 2:**
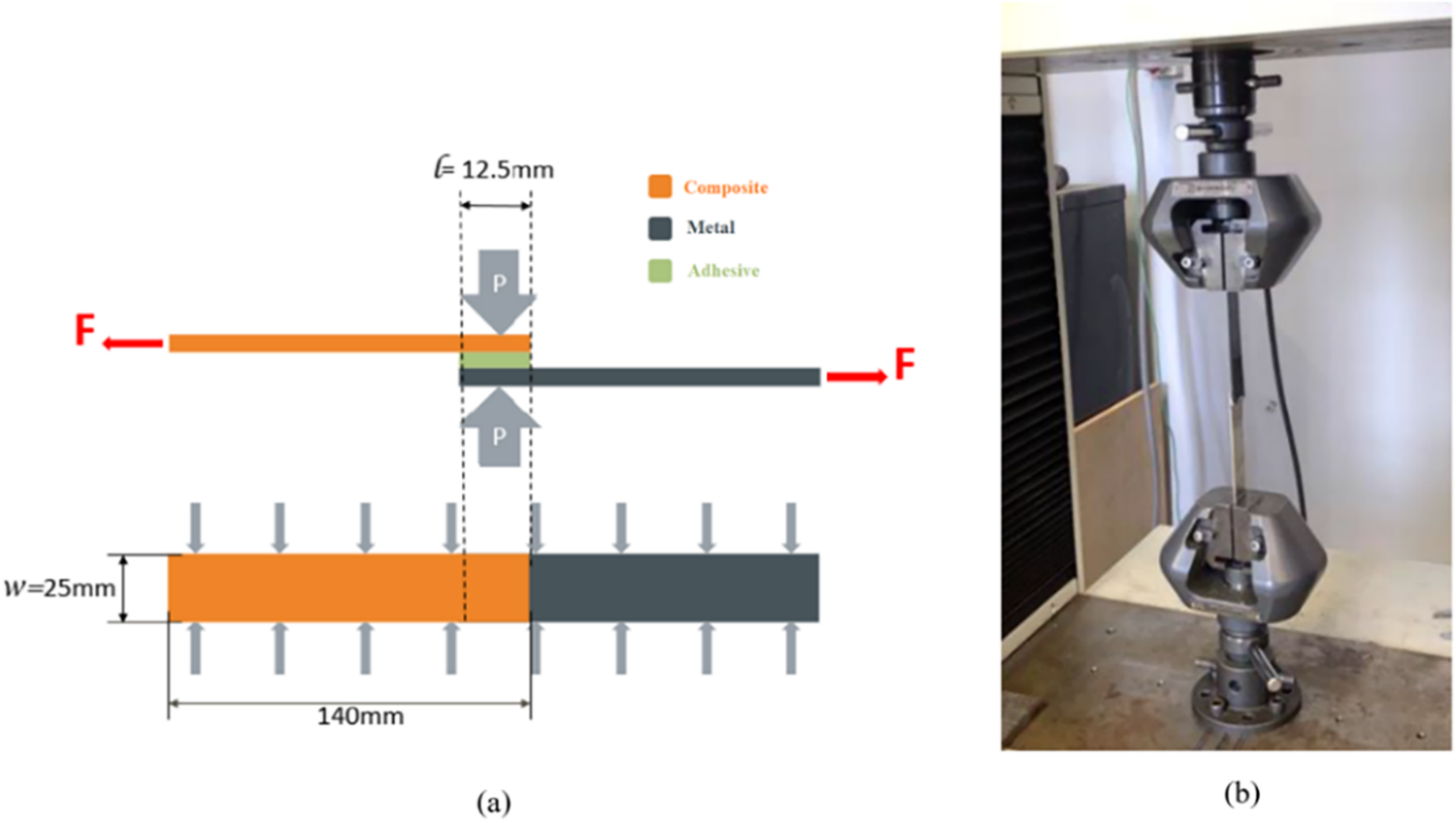
(a) Representation of the adhesive-bonded hybrid joint. (b) Test setup.

### 2.3 Numerical Study: Cohesive Zone Modeling (CZM)

The finite element model of the single-layer joint was prepared in ABAQUS/CAE (SIMULIA by Dassault Systems), and a nonlinear analysis was performed using Abaqus/Standard. A 3D model was used to simulate the out-of-plane stress distribution and bending stresses generated in a single-bonded joint. The upper and lower parts of the joint, the adhesive, and the adhesive interfaces were modeled as a whole.

Cohesive elements were carried out for the adhesive, and continuum shell elements were performed for the bonded parts. For modeling the bonded parts, eight-node continuous shell elements (S8CR) with reduced integration were conducted. For the adhesive, eight-node cohesive elements with 4 integration points (COH3D8) were used. Also, the boundary conditions were simulated in the jaws of the tensile testing machine. An exponential behavior was performed for the adhesive in the traction-separation law. The quadratic nominal stress criterion (QUADS) was selected as the damage initiation criterion because it assumes a stress relationship that considers different directions. The critical energy release rate was chosen as the power law mixed-mode crack growth criterion. The material specifications of adhesive for cohesive zone modeling are given in Table 2.

**Table 2:** Material Specifications of the Adhesive for CZM

adhesive	*E*_1_	*E*_2_	*E*_3_	*t*_n_	*t*_s_	*t*_t_	*G*_IC_	*G*_IIC_	*G*_IIIC_
Hysol Loctite EA 9394	2.7 GPa	1 GPa	1 GPa	46 MPa	28.9 MPa	28.9 MPa	100 J/mm^2^	160 J/mm^2^	160 J/mm^2^

*E*_1_–*E*_3_ define the initial elastic stiffness of the adhesive; *t*_n_, *t*_s_, and *t*_t_ represent the maximum nominal stresses at damage initiation; and *G*_IC_, *G*_IIC_ and *G*_IIIC_ correspond to the critical fracture energies. The manufacturer’s data provided the fracture energy values (*G*_IC_, *G*_IIC_, and *G*_IIIC_), which were then calibrated to correspond with the experimental load–displacement response and peak load values. By calibrating the cohesive parameters, the effective interfacial behavior under various surface conditions is guaranteed. Accounting for variations in interfacial behavior resulting from material qualities and surface conditions, the cohesive parameters were calibrated independently for every combination of materials. Moreover, a mesh sensitivity study was carried out for identification of productive analysis conditions.

## 3 Results and Discussion

### 3.1 Single-Lap Shear Test Results

Previous studies have shown that increasing the contact between the surface area and the bonded surfaces will increase the number of polymer bonds, thereby increasing the strength. To observe this in the bonds, different surface roughening techniques were applied to the surface, with the aim of increasing the contact between the surfaces.

The surface roughness (*R*_a_) was measured by using an optical profilometer Mitutoyo SJ 220, and similar roughness levels were obtained for all specimens after surface preparation. In the baseline untreated surface, all materials exhibited relatively low roughness values. The *R*_a_ values were measured as 0.4 μm for Al6061, 1.4 μm for SS304, 0.4 μm for Ti6Al4 V, and 0.55 μm for PA66. Following sandblasting, a noticeable increase in the surface roughness was observed. The *R*_a_ values increased to 4.6 μm for Al6061, 1.25 μm for SS304, 0.73 μm for Ti6Al4 V, and 7.3 μm for PA66. After sandblasting, the roughness further increased, reaching 8.8 μm for Al6061, 6.4 μm for SS304, 4.65 μm for Ti6Al4 V, and 5.2 μm for PA66. The laser surface texturing treatment produced the highest roughness values among all of the conditions. *R*_a_ values of 14, 12.5, and 13.3 μm were obtained for Al6061, SS304, and Ti6Al4 V, respectively.

Changes in the material hardness can also account for the changes in the surface roughness that are obtained following surface treatments. In contrast to softer materials, harder materials typically show more localized deformation during mechanical treatments, leading to distinct roughness profiles.^2^ For instance, because of their varied mechanical characteristics, titanium and stainless steel react differently to abrasive and blasting operations, which helps explain the observed differences in roughness levels. The lap shear strength comparisons of hybrid joints according to different surface roughening methods are shown in Figure 3.

**Figure 3:**
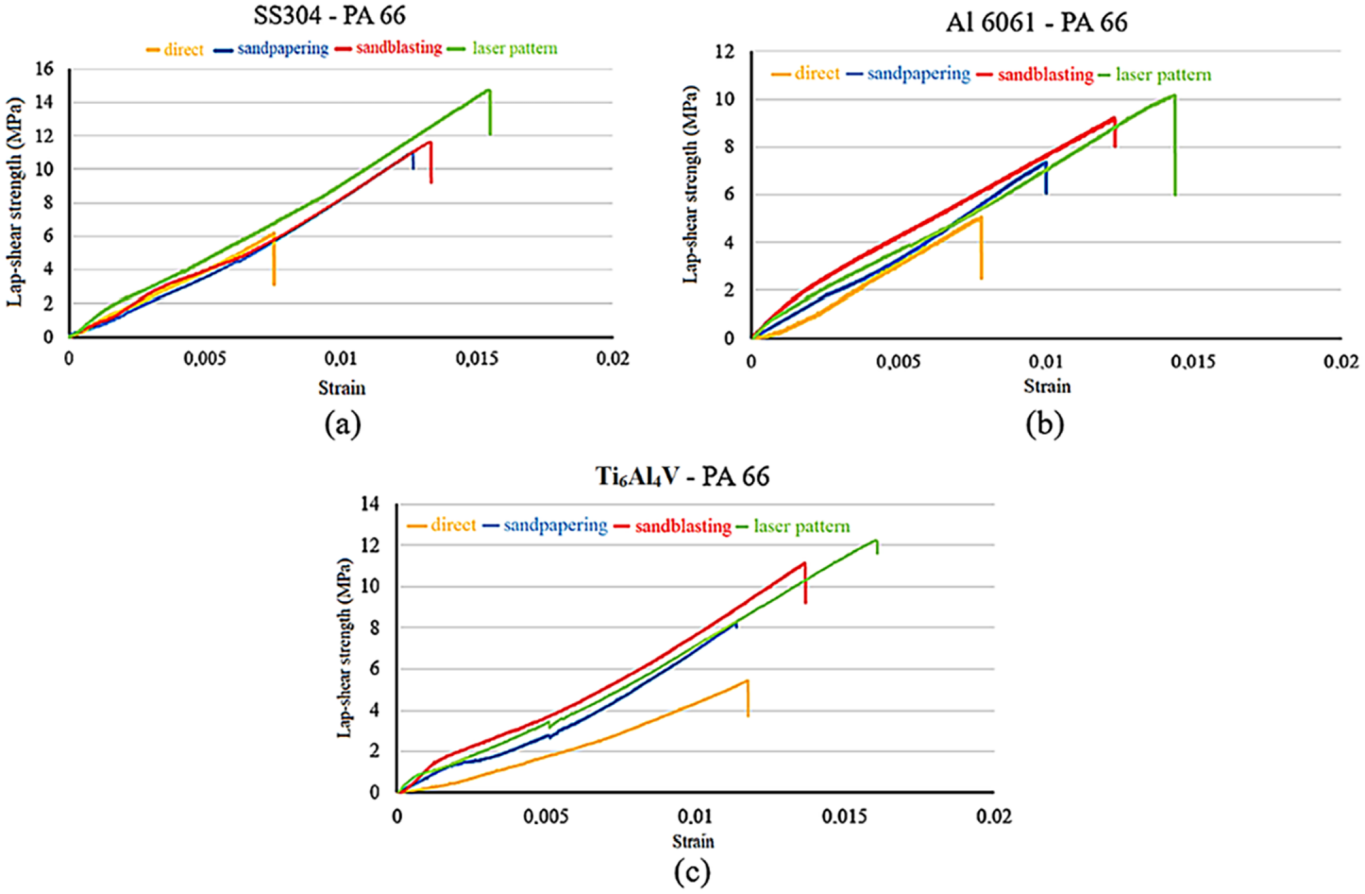
Lap shear strength-strain of surfaces for all surface roughness conditions: (a) SS304 and PA66, (b) Al6061 and PA66, and (c) Ti_6_Al_4_V and PA66.

As seen in Figure 3a, the highest shear strength of the adhesive bond was achieved with laser surface texturing after sandblasting at 14.7 ± 0.18 MPa with an SS304 and PA66 hybrid joint. Abrasive sanding surfaces increased the shear strength of the bond by 80%, from 6.1 ± 0.05 to 11 ± 0.1 MPa, while sandblasting the surfaces increased the shear strength to 11.8 ± 0.12 MPa, a 90% increase. Laser surface texturing after sandblasting of the surfaces increased the shear strength to 14.7 ± 0.18 MPa, representing a 141% increase compared to the plain surface.

Also, in Figure 3b, the highest strength of 10.1 ± 0.09 MPa was reached in the Al6061 and PA66 hybrid joint with the laser surface texturing application after sandblasting. By abrasive sanding the surface, the connection shear strength increased from 5 ± 0.05 to 7.2 ± 0.07 MPa, representing a 44% increase, while sandblasting the surfaces increased the shear strength to 9.1 ± 0.08 MPa, performing an 82% increase. Laser surface texturing after sandblasting of the surfaces increased the shear strength to 10.1 ± 0.09 MPa, representing a 102% increase compared to the plain surface.

The highest lap shear strength of 12.2 ± 0.15 MPa in the Ti_6_Al_4_V-PA66 hybrid joint was measured with the laser surface texturing technique after sandblasting. Abrasive sanding the surfaces increased the shear strength of the joint from 5.4 ± 0.06 to 8.1 ± 0.08 MPa, while sandblasting the surfaces increased the lap shear strength twice. By processing the surface with a laser surface texturing after sandblasting, the shear strength increased to 12.2 ± 0.15 MPa.

It is observed in Figure 4a that damage in the hybrid bond between the surface obtained by abrasive sanding, sandblasting, and laser surface texturing after sandblasting on the stainless-steel material surface and the baseline untreated surface with GF-reinforced PA66/SS304 starts from both surfaces. The adhesive was broken down and remained on both surfaces under all surface conditions. This indicates that the surface was mechanically and chemically well prepared. Also, the surfaces of baseline untreated surfaces exhibit predominantly adhesive failure, owing to clean substrate areas and limited adhesive residue.

**Figure 4:**
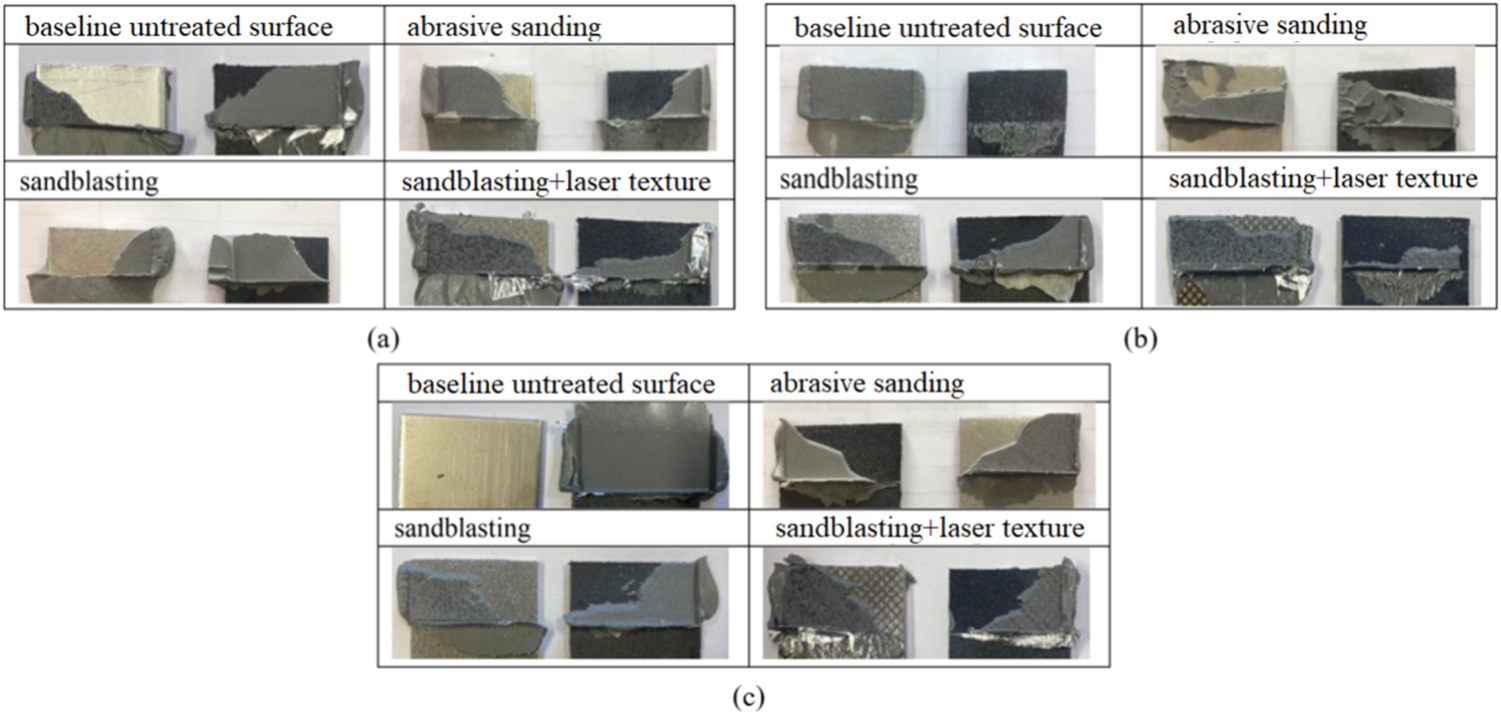
Damaged surfaces of all surface roughness conditions for (a) SS304 and PA66, (b) Al6061 and PA66, and (c) Ti_6_Al_4_V and PA66 hybrid joints.

In Figure 4b, surface damage figures are visible in the hybrid joint between GF PA66/Al6061 and the surface obtained by abrasive sanding, sandblasting, and laser surface texturing on the sandblasted surface and the baseline untreated surface operation. Except for the baseline untreated surface operation, the adhesive was broken down and remained on both surfaces under all surface conditions. In the baseline untreated surface conditions, the adhesive remained on the metal surface. The fact that the adhesive remains entirely on one surface indicates that the adhesive bond becomes stronger than the cohesive bond. This is because the surface did not achieve a sufficient roughness. Moreover, the abrasive sanding and sandblasted surfaces represent mixed adhesive-cohesive failure, with adhesive distributed on both adherends, indicating improved interfacial bonding.

Figure 4c shows postdamage images of the hybrid bond between PA66 and Ti_6_Al_4_V the surface obtained by abrasive sanding, sandblasting, and laser surface texturing on the sandblasted surface, as well as the baseline untreated surface. Except for the baseline untreated surface, the adhesive was fragmented and remained on both surfaces in all surface conditions. The laser surface texturing after sandblasting results mainly in cohesive failure within the adhesive layer, as evidenced by extensive adhesive remnants and tearing, suggesting that the interfacial bond strength exceeds the cohesive strength of the adhesive. The lap shear test conducted on metal-composite joint surfaces revealed that increasing the roughness of all composite and metal surfaces improved adhesion performance and increased joint strength. Additionally, the laser surface texturing after sandblasting yielded the best results, with the highest lap shear strength observed between GF PA66/SS304. The lowest lap shear strength was measured between GF PA66/Al6061.

### 3.2 Effect of Temperature on Lap Shear Strength

The adhesive bonds made with structural adhesives (mainly epoxies) showed a decrease in strength with increasing and decreasing temperatures. When the temperature exceeds the glass transition temperature of the materials, the lap shear strength of adhesives drops. The glass transition temperature of the Hysol Loctite 9394 adhesive used in the study is 74 °C. Above the glass transition temperature, the adhesive behaves viscously, and microcracks form more easily in the bonding area than at room temperature. Consequently, at high temperatures, the adhesive softens and acts weakly to sustain the load. At low temperatures, shrinkage occurs in the adhesives, and this shrinkage causes excessive stresses in the adhesive. As a result, at low temperatures, the strength of the bond decreases due to high thermal stresses and increased brittleness of the adhesive. For identification of adhesive strength at different temperatures, the specimen was stored in a climate chamber at −40 °C for 24 h and at 85 °C for 48 h according to ISO 16750-4.

Lap shear strength-strain of SS304-PA66 hybrid joints for different temperatures and different conditions can be seen in Figure 5. A general decrease in the shear strength of SS304-PA66 hybrid joints at different temperatures compared to room temperature was observed. The quality of the interfacial bonding has a significant impact on how temperature affects lap shear strength. Low temperatures (−40 °C) make adhesives more brittle and encourage early interfacial breakdown on baseline untreated surfaces, where adhesion is dominated by weak interfacial bonding (Figure 5a). Stress transfer is enhanced by improved mechanical interlocking for mechanically treated surfaces, such as abrasive sanded and laser-textured specimens. In these circumstances, the adhesive’s higher strength is a result of its increased stiffness at low temperatures.

**Figure 5:**
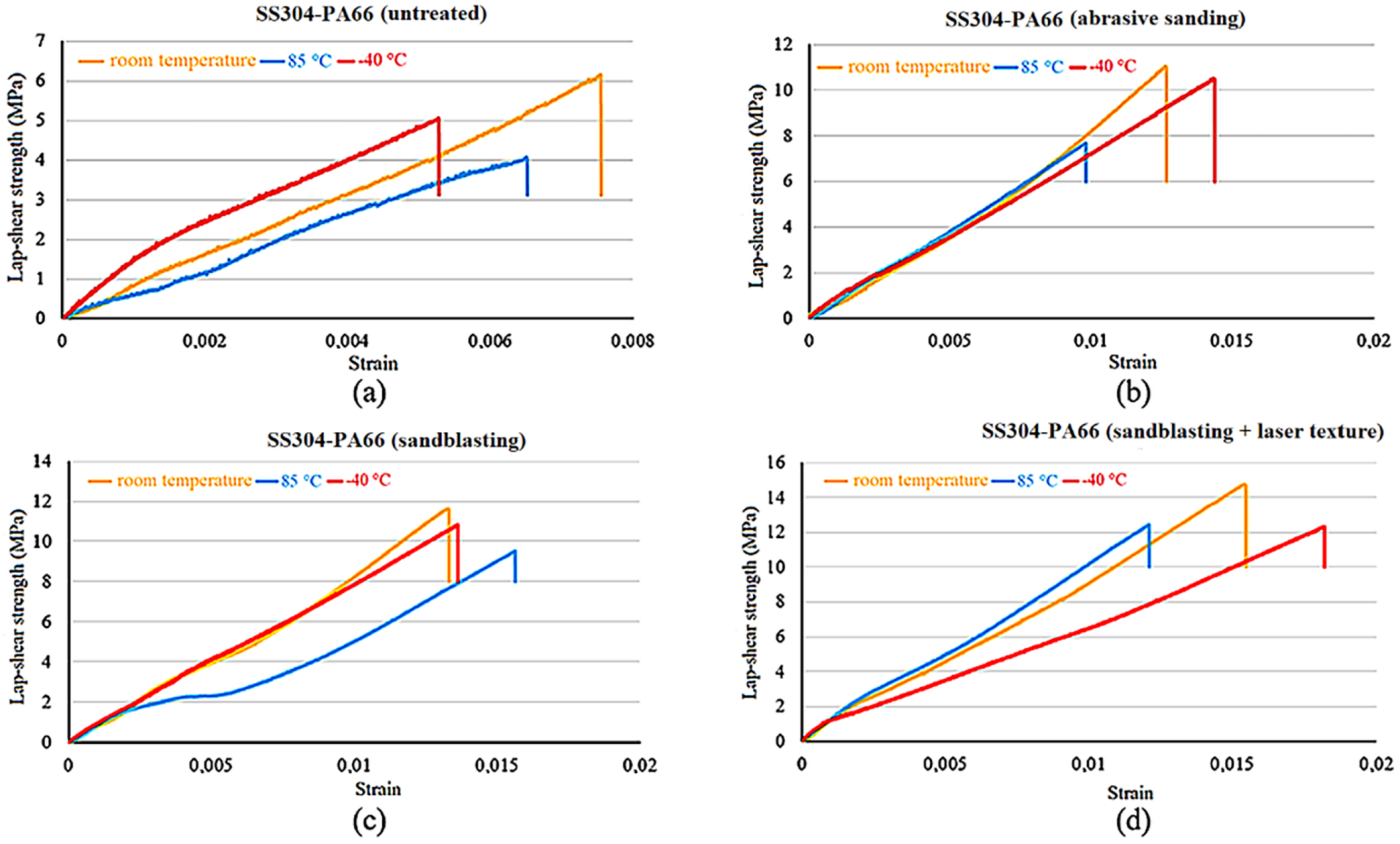
Lap shear strength-strain of SS304-PA66 hybrid joints for (a) baseline untreated surface, (b) abrasive sanding, (c) sandblasting, and (d) laser surface texturing after sandblasting.

On the other hand, high temperatures weaken the adhesive layer, which lowers strength and decreases load transfer efficiency (Figure 5b,d). Among the joints tested at 85 °C, the highest decrease compared to the shear strength of joints at room temperature occurred in the sandblasting technique, with a decrease of approximately 25%. In joints tested at −40 °C, a decrease of approximately 20% occurred in the baseline untreated surface.

Lap shear strength-strain of Al6061-PA66 hybrid joints for different temperatures and different conditions can be seen in Figure 6. A general decrease in the shear strength of Al6061-PA66 hybrid joints was observed at different temperatures compared with room temperature. Among the joints tested at 85 °C, the highest decrease in shear strength compared to room temperature joints occurred in the sandblasting operation, with a decrease of approximately 26%. In joints tested at −40 °C, a decrease of approximately 23% occurred on the sandblasted surface.

**Figure 6:**
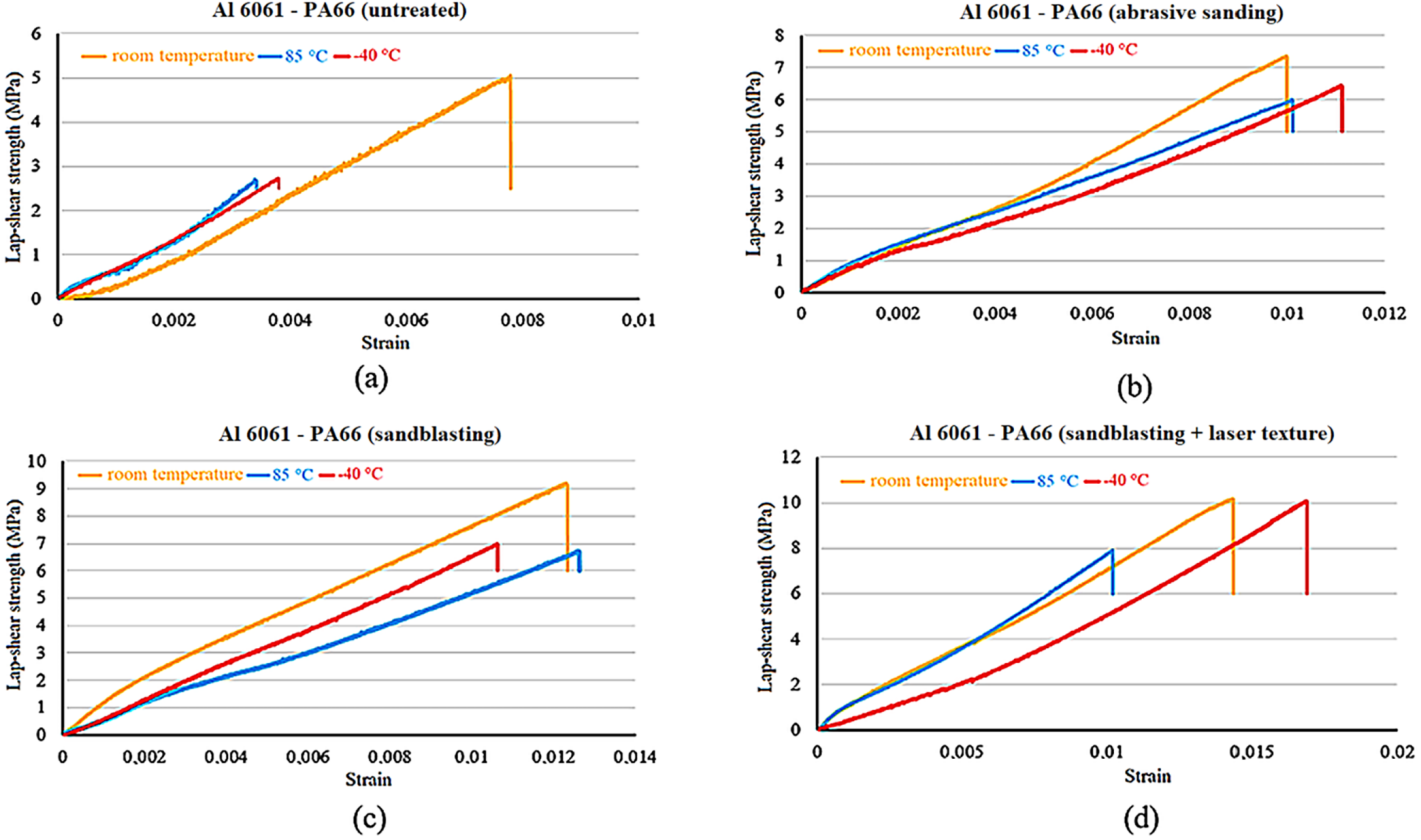
Lap shear strength-strain of Al6061-PA66 hybrid joints for (a) baseline untreated surface, (b) abrasive sanding, (c) sandblasting, and (d) laser surface texturing after sandblasting.

Lap shear strength-strain of Ti_6_Al_4_V-PA66 hybrid joints for different temperatures and different conditions can be seen in Figure 7. A general decrease in the lap shear strength of Ti_6_Al_4_V-PA66 hybrid joints at different temperatures compared with room temperature was observed. Among the joints tested at 85 °C, the highest decrease in lap shear strength compared to room temperature joints occurred in the abrasive sanding technique, with a decrease of approximately 36%. In joints tested at −40 °C, a decrease of approximately 45% occurred on the baseline untreated surface.

**Figure 7:**
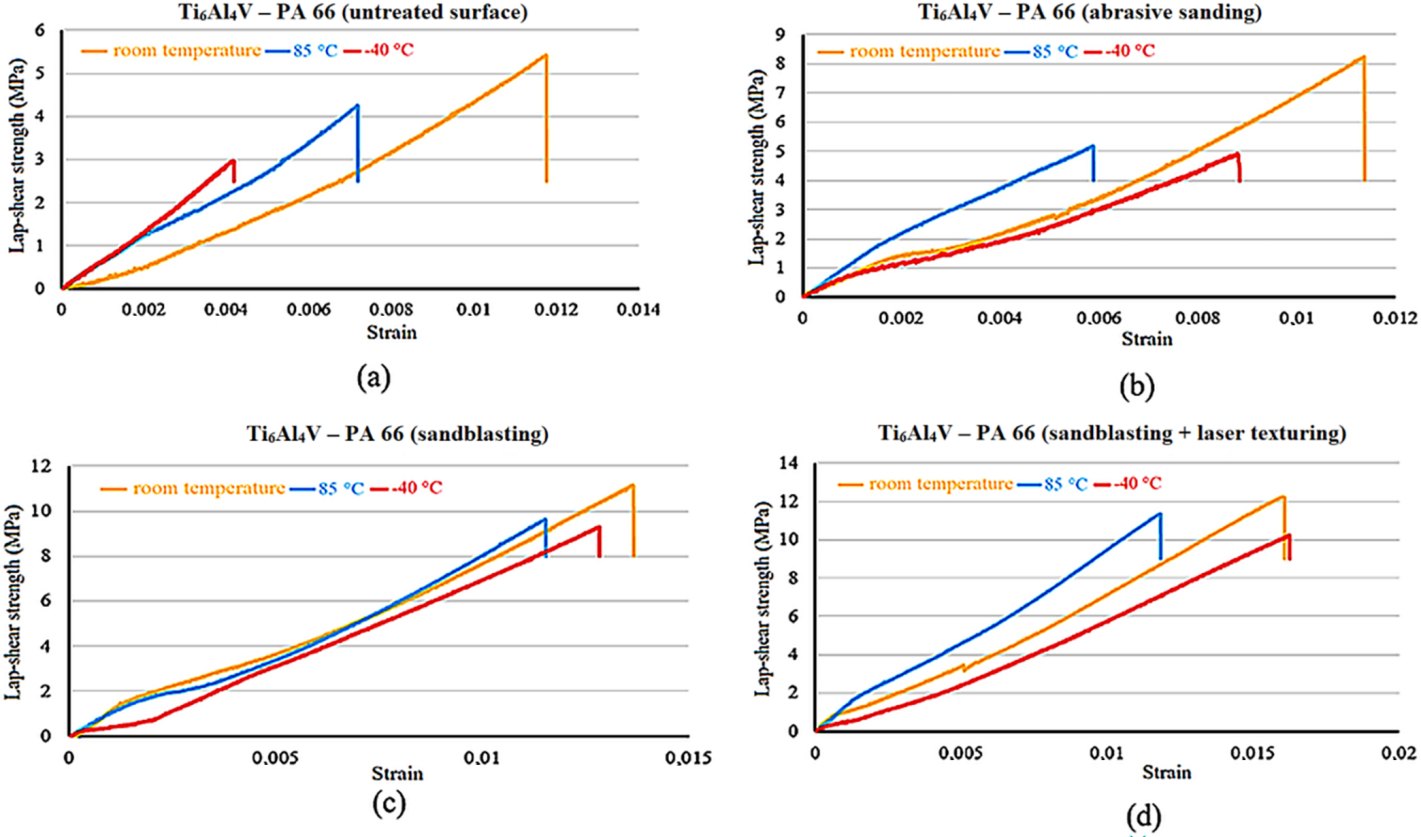
Lap shear strength-strain of Ti_6_Al_4_V-PA66 hybrid joints for (a) baseline untreated surface, (b) abrasive sanding, (c) sandblasting, (d) laser surface texturing after sandblasting.

Figure 8a,b shows the damage micrographs of the hybrid adhesive bond between stainless steel material and GF-reinforced PA at −40 and +85 °C under all surface conditions. In tests conducted at +85 °C, the adhesive remained on both surfaces on the baseline untreated surface and sandblasted surfaces, while on surfaces with laser surface texturing applied over sandblasting and abrasive sanding, the adhesive remained on only one surface. This can be explained by the adhesive becoming more ductile as the temperature exceeds the transition temperature of the material. In tests conducted at −40 °C, the adhesive remained on only one surface under all conditions except for surfaces treated with sandblasting. The adhesive remaining on only one surface can be explained by the adhesive becoming brittle and its internal stresses increasing at low temperatures.

**Figure 8:**
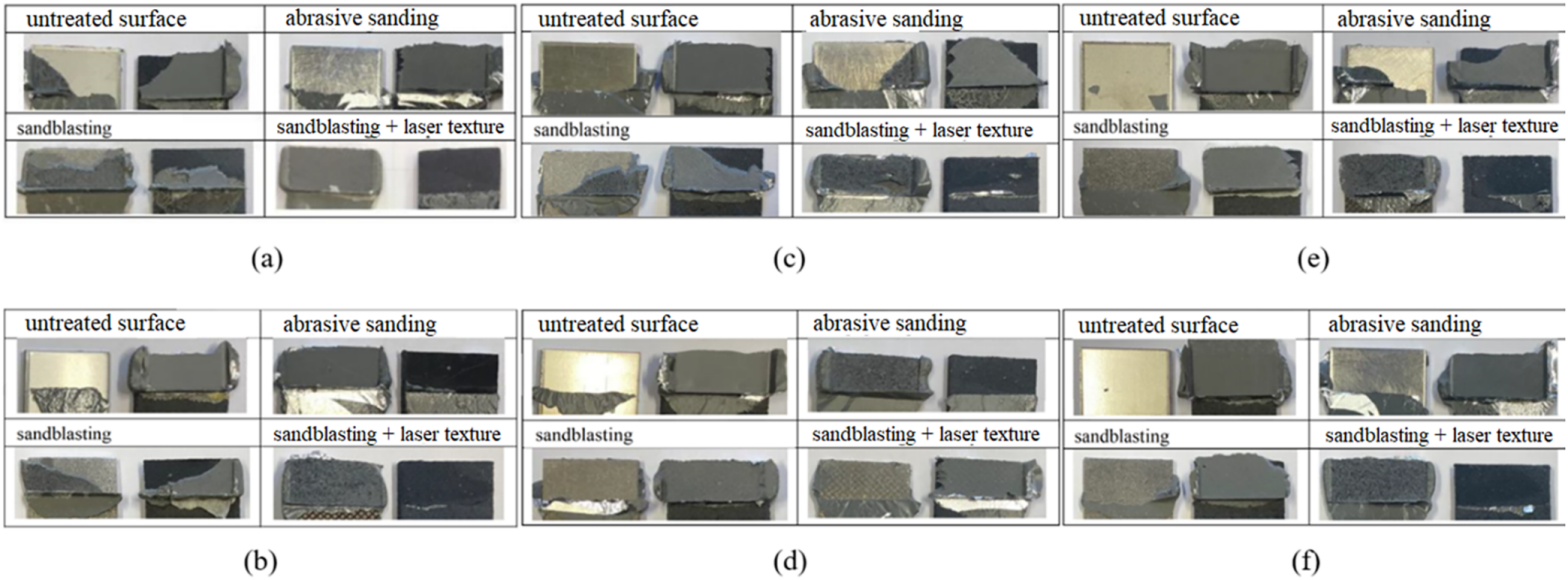
(a) Damage surfaces of the SS304-PA66 joint at +85 °C. (b) Damage surfaces of the SS304-PA66 joint at −40 °C. (c) Damage surfaces of the Al6061-PA66 joint at +85 °C. (d) Damage surfaces of the Al6061-PA66 joint at −40 °C. (e) Damage surfaces of the Ti_6_Al_4_V-PA66 joint at +85 °C. (f) Damage surfaces of the Ti_6_Al_4_V-PA66 joint at −40 °C.

Figure 8c,d represents the damage micrographs of the hybrid adhesive bond between aluminum material and GF-reinforced PA at −40 and +85 °C under all surface conditions. In tests conducted at +85 °C, the adhesive remained on both surfaces when abrasive sanding and sandblasting processes were applied to the surfaces, while on the baseline untreated surface and surfaces with laser surface texturing applied over sandblasting, the adhesive remained on only one surface. In tests conducted at −40 °C, the adhesive remained on only one surface under all surface conditions. This indicates that the surface roughening processes applied at −40 °C need to be improved.

Figure 8e,f gives the damage micrographs of the hybrid adhesive bond between titanium material and GF-reinforced PA at −40 and +85 °C under all surface conditions. In tests conducted at +85 °C, the adhesive remained on both surfaces when abrasive sanding was applied, while on all other surfaces, the adhesive remained on only one surface. In tests conducted at −40 °C, the adhesive remained on only one surface under all surface conditions. At −40 °C, the adhesive becomes quite brittle and completely separates from the surface.

Also, Table 3 compares the effects of surface preparation techniques on the lap shear strength, while Table 4 shows the effect of temperature differences on the lap shear strength. The tests conducted proved that surface roughness enhances the adhesive bonding of the hybrid joint, thereby increasing its lap shear strength. The difference in surface roughness obtained on different metal types as a result of the same surface roughening processes applied to the material surfaces is due to the difference in hardness between the metals.

**Table 3:** Effect of Surface Preparation on Lap Shear Strength

specimen		baseline untreated surface	abrasive sanding	sandblasting	sandblasting + laser surface texturing
SS304-PA 35% GF	τ_max_ (MPa)	5 ± 0.05	10.3 ± 0.1	11 ± 0.11	12.1 ± 0.13
–40 °C	% difference		106	120	142
SS304-PA 35% GF	τ_max_ (MPa)	6.1 ± 0.05	11 ± 0.1	11.8 ± 0.12	14.7 ± 0.18
25 °C	% difference		80.33	93.44	140.98
SS304-PA 35% GF	τ_max_ (MPa)	4 ± 0.04	7.5 ± 0.07	9.5 ± 0.09	12.3 ± 0.11
85 °C	% difference		87.5	137.5	207.5
Al6061 T6-PA 35% GF	τ_max_ (MPa)	2.7 ± 0.03	6.5 ± 0.06	7 ± 0.09	10 ± 0.1
–40 °C	% difference		140.74	159.26	270.37
Al6061 T6-PA 35% GF	τ_max_ (MPa)	3 ± 0.04	7.2 ± 0.07	9.1 ± 0.08	10.1 ± 0.09
25 °C	% difference		140	203.33	236.67
Al6061 T6-PA 35% GF	τ_max_ (MPa)	2.7 ± 0.03	6 ± 0.05	6,7 ± 0.06	8 ± 0.07
85 °C	% difference		122.22	148.15	196.3
Ti_6_Al_4_V-PA 35% GF	τ_max_ (MPa)	3 ± 0.04	5 ± 0.04	9.1 ± 0.09	10.1 ± 0.1
–40 °C	% difference		66.67	203.33	236.67
Ti_6_Al_4_V-PA 35% GF	τ_max_ (MPa)	5.4 ± 0.06	8.1 ± 0.08	11 ± 0.1	12.2 ± 0.15
25 °C	% difference		50	103.7	125.93
Ti_6_Al_4_V-PA 35% GF	τ_max_ (MPa)	4.2 ± 0.05	5.1 ± 0.05	9.5 ± 0.1	11.2 ± 0.14
85 °C	% difference		21.43	126.19	166.67

**Table 4:** Effect of Temperature on Lap Shear Strength

surface preparation		–40 °C	25 °C	85 °C
baseline untreated surface	τ_max_ (MPa)	5 ± 0.05	6.1 ± 0.05	4 ± 0.04
	% difference	18.03		34.43
abrasive sanding	τ_max_ (MPa)	10.3 ± 0.1	11 ± 0.1	7.5 ± 0.07
	% difference	6.36		31.82
sandblasting	τ_max_ (MPa)	11 ± 0.11	11.8 ± 0.12	9.5 ± 0.09
	% difference	6.78		19.49
sandblasting + laser surface texturing	τ_max_ (MPa)	12.1 ± 0.13	14.7 ± 0.18	12.3 ± 0.11
	% difference	6.78		16.33
baseline untreated surface	τ_max_ (MPa)	2.7 ± 0.03	3 ± 0.04	2.7 ± 0.03
	% difference	10		10
abrasive sanding	τ_max_ (MPa)	6.5 ± 0.06	7.2 ± 0.07	6 ± 0.05
	% difference	9.72		16.67
sandblasting	τ_max_ (MPa)	7 ± 0.09	9.1 ± 0.08	6.7 ± 0.06
	% difference	23.08		26.37
sandblasting + laser surface texturing	τ_max_ (MPa)	10 ± 0.1	10.1 ± 0.09	8 ± 0.07
	% difference	1		20.79
baseline untreated surface	τ_max_ (MPa)	3 ± 0.04	5.4 ± 0.06	4.2 ± 0.05
	% difference	44.44		22.22
abrasive sanding	τ_max_ (MPa)	5 ± 0.04	8.1 ± 0.08	5.1 ± 0.05
	% difference	38.27		37.04
sandblasting	τ_max_ (MPa)	9.1 ± 0.09	11 ± 0.1	9.5 ± 0.1
	% difference	17.27		13.64
sandblasting + laser surface texturing	τ_max_ (MPa)	10.1 ± 0.1	12.2 ± 0.15	11.2 ± 0.14
	% difference	17.21		8.2

Laser surface texturing geometrically optimizes the mechanical interlocking at the adhesive-surface interface, creating a clean, high-energy surface that allows the adhesive to completely wet the surface and penetrate microvoids, thereby improving stress distribution and enhancing chemical bonding. This combination provides higher single-lap joints (SLJ) strength compared with abrasive sanding and sandblasting.

Owing to surface chemistry and wettability, which encourage efficient adhesive spreading and interfacial bonding, they are responsible for the improved lap shear strength seen for SS304 adherends. Stable oxide coatings on stainless steel surfaces facilitate robust adhesive bonds. Conversely, Ti_6_Al_4_V adherends showed less strength, most likely because of a persistent titanium oxide layer that can inhibit adhesive wetting and diminish surface reactivity. These oxide layers could limit the strength of the interfacial connection by acting as weak boundary layers.^23^ Variations in the surface morphology and mechanical interlocking following surface treatment could possibly be a factor in the observed differences. The findings show that the surface shape and interfacial chemistry are important factors in determining joint strength.

### 3.3 Results of Cohesive Zone Modeling

The time-dependent stress distribution of the single-layered joint cohesive zone model solved with the Abaqus program is shown in Figure 9. When examining the stress distribution on the model, it is observed that high stress values occur at the adhesive corners due to singularities. As the stress continues to increase, the adhesive begins to separate from the metal and composite surfaces starting from the corners. This separation continues until the bond is completely broken. As a result of the breakage, the adhesive is completely destroyed and the surfaces separate from each other.

**Figure 9:**
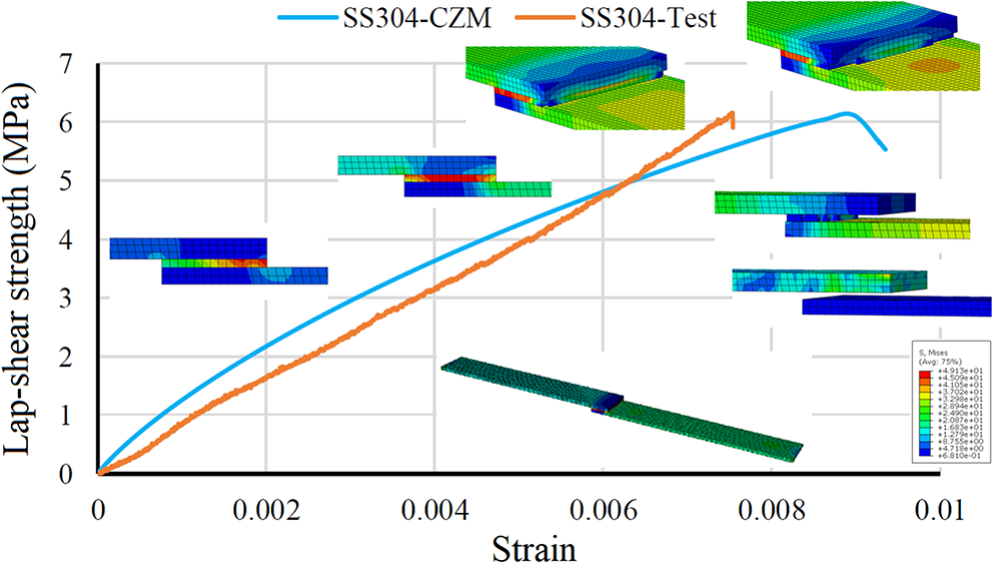
Failure process and stress distribution of a single-layer joint under time-dependent increasing stress.

The study for determining the optimal mesh size in the CZM study within the bonding work involved a parametric mesh sensitivity study using five different mesh sizes (1, 0.5, 0.35, 0.2, and 0.1 mm) in the interface modeling to include estimates close to the maximum load and to enable an efficient analysis study. The maximum load experienced by the connection was compared with mesh refinement, as shown in Figure 10. The maximum lap shear stress obtained with a mesh size of 0.2 mm is 5.4 MPa, while the result for the finer 0.1 mm mesh is 5.3 MPa, corresponding to a difference of lower than 2%. Since the 0.1 mm mesh size required a very long calculation time, cohesive elements were meshed with 0.2 mm elements, which yielded a result very close to the maximum load and was therefore preferred.

**Figure 10:**
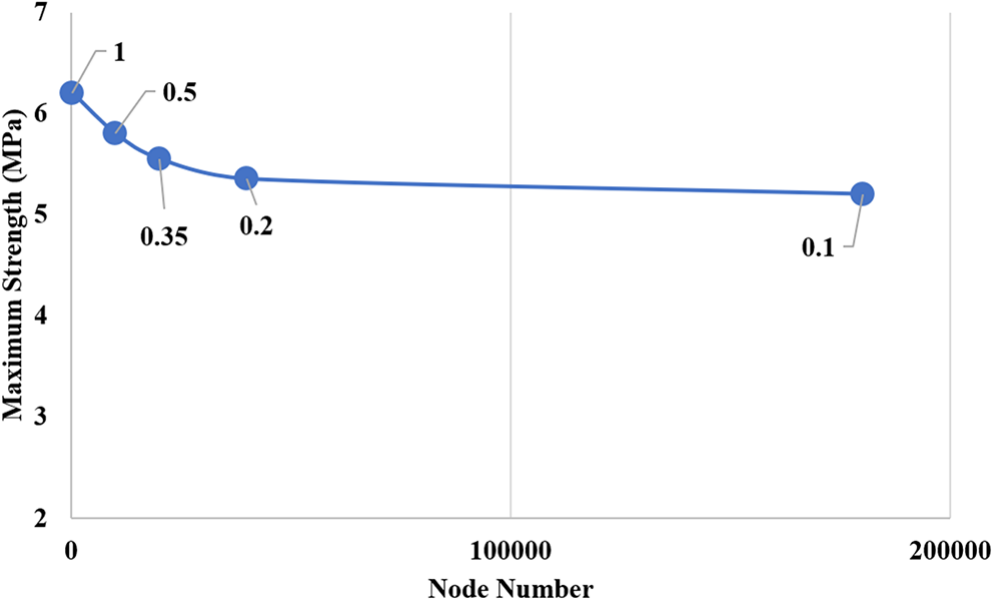
Mesh sensitivity.

The lap shear strength obtained from the cohesive zone model for each of the three metallic materials is listed in Figure 11a. Also, the differences between the test results and the finite element model obtained are shown graphically in Figure 11b–d. When examined in three different materials, the cohesive zone model yielded results that were largely similar to those obtained in experimental tests. The highest similarity was achieved in the SS304 material, with a difference of only 1.5%. The least similarity was achieved in Ti_6_Al_4_V material, with a difference of 15%. Although all three materials yield similar finite element results, the surface preparations applied increase lap shear strength, and particularly in stainless steel materials, lap shear strength increases by 2–3 times after surface preparation. The cohesive zone parameters were calibrated using nominal material properties, which may not fully capture the interface behavior for titanium adherends.

**Figure 11:**
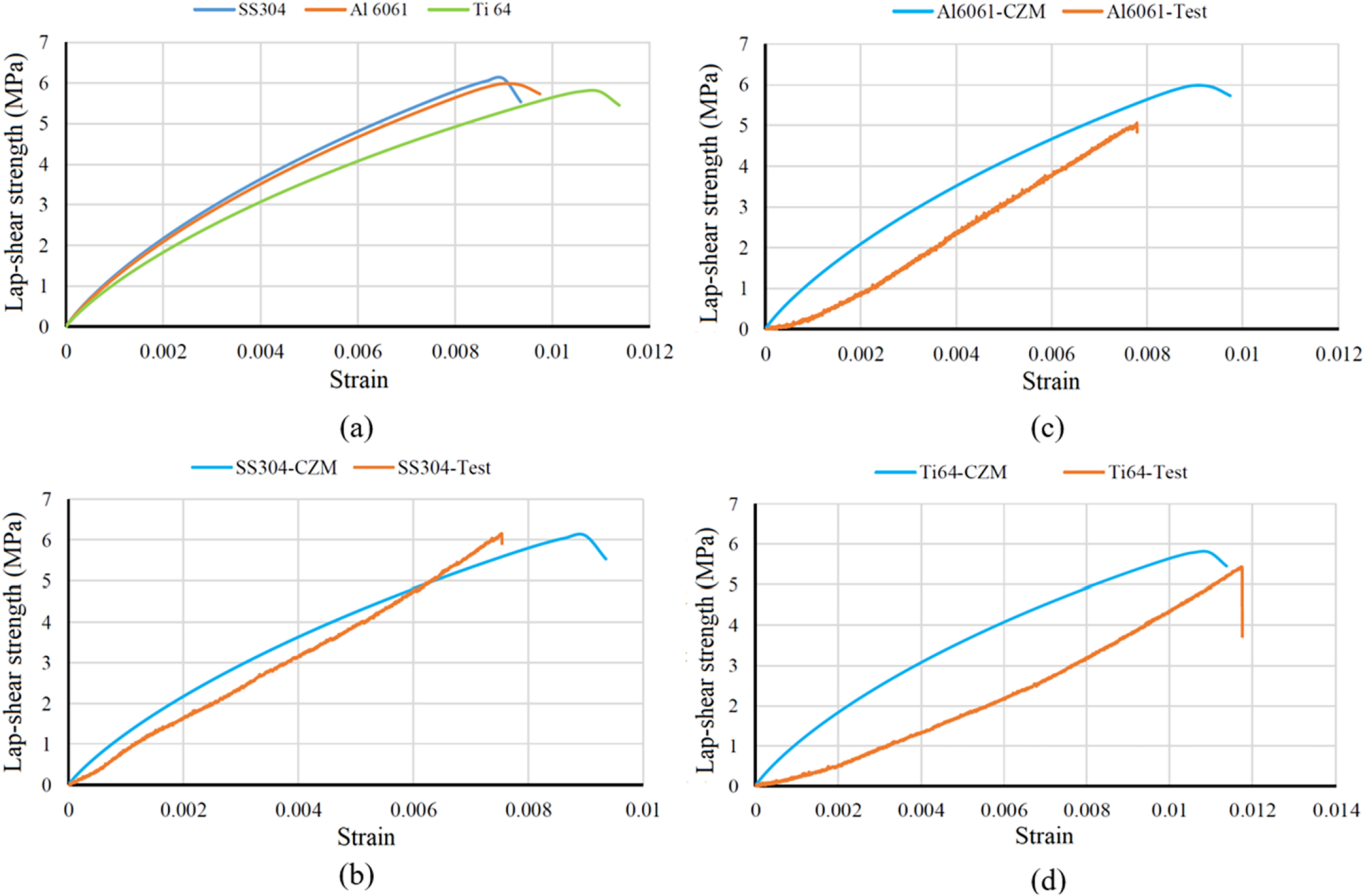
Lap-shear strength obtained from the cohesive zone model for each of the three metallic materials. (a) All three materials, (b) Al6061, (c) SS304, (d) Ti_6_Al_4_V.

The greater deviation between simulation and experimental results for the Ti_6_Al_4_V SLJ compared to Al6061 and AISI 304 joints can mainly be owed to physiochemical characteristics of the titanium alloy surface and its high stiffness, which creates a complex stress concentration. Besides, the differences in surface energy and adhesion characteristics and potential variations in surface preparation sensitivity may affect the results. These are inherently challenging to model accurately in an adhesive joint. Titanium forms an oxide layer on the surface and it completely affects the interfacial bond strength.^23^ Despite this variation, the model successfully captures the overall lap shear strength–strain response and failure behavior trends for both material systems.

## 4 Conclusions

This study thoroughly demonstrated the critical effect of different surface preparation methods on the mechanical performance and interfacial behavior when bonding a 35% glass-fiber-reinforced polyamide thermoplastic composite material to three different metallic materials, namely, stainless steel, aluminum, and titanium. Experimentally, it was observed that the four different surface treatments applied created a significant difference in the bond strength, as measured by lap shear tests. The surface treatments yielding the highest lap shear strength values were, in order, laser surface texturing after sandblasting, sandblasting, abrasive sanding, and a baseline untreated surface. Moreover, each fracture surface was examined in detail optically. The materials were ranked in order of lap shear strength as stainless steel (SS304), aluminum (Al6061), and finally titanium (Ti_6_Al_4_V). The Cohesive Zone Model (CZM), developed to model interfacial behavior, yielded results that were highly consistent with experimental data 1.5% with Al6061/GF-reinforced PA; it was confirmed that the model can reliably predict the stress–strain relationship and damage progression at the interface for different metal types. This strong correlation between experimental tests and the numerical model demonstrates that the developed CZM can be used as a reliable prediction tool in the design and optimization processes of similar hybrid structures. The modeling framework’s validity is confirmed by the low error levels and steady correlation with experimental results. All things considered, the bilinear cohesive zone model correctly forecasts the beginning and spread of the adhesive layer deterioration. The traction-separation law and related parameters can accurately represent the mechanical response of the bonded joints, as shown by the strong agreement between the computational and experimental data. Also, the consistency between numerical and experimental curves further supports the reliability of the model. As a result, this research emphasizes that not only the type of metal but also the selection of an appropriate surface preparation method are equally important in optimizing adhesive bonding, and it provides both an experimental and numerical methodological framework for future hybrid material combinations.
